# *N*-(4-Hydroxy­phen­yl)-2-(1,1,3-trioxo-2,3-di­hydro-1,2-benzothia­zol-2-yl)­acet­amide

**DOI:** 10.1107/S1600536809023022

**Published:** 2009-06-24

**Authors:** Abha Verma, Edwin D. Stevens

**Affiliations:** a Department of Chemistry, University of New Orleans, New Orleans, LA 70148, USA

## Abstract

In the title compound, C_15_H_12_N_2_O_5_S, the benzisothia­zole group is approximately planar (r.m.s. deviation excluding H atoms and the two O atoms bonded to S = 0.023 Å). The dihedral angle between the benzisothia­zole ring and the terminal phenol ring is 84.9 (1)°. In the crystal, mol­ecules are joined by N—H⋯O and O—H⋯O hydrogen bonds, and π-stacking inter­actions are observed between alternating phenol and benzisothia­zole rings [centroid–centroid distances = 3.929 (3) and 3.943 (3) Å].

## Related literature

For background literature related to analgesics, see: Slattery *et al.* (1996 ▶); McGoldrick & Bailie (1997 ▶); Watkins *et al.* (2006 ▶). For the synthesis and biological activity of the title compound, see: Vaccarino *et al.* (2007 ▶); González-Martin *et al.* (1998 ▶); Bazan & Alvarez-Builla (1996*a* ▶,*b* ▶). For related structures, see: Arshad *et al.* (2009**a* ▶,*b* ▶,c* ▶); Siddiqui *et al.* (2008**a* ▶,b* ▶; 2007 ▶).
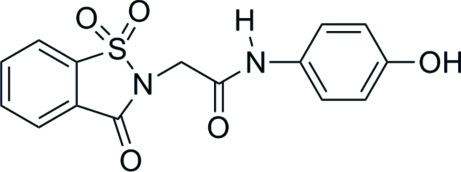


## Experimental

### 

#### Crystal data

C_15_H_12_N_2_O_5_S
*M* *_r_* = 332.33
Orthorhombic, 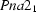

*a* = 16.3588 (10) Å
*b* = 9.6451 (6) Å
*c* = 9.9603 (6) Å
*V* = 1571.56 (17) Å^3^
*Z* = 4
Mo *K*α radiation
μ = 0.23 mm^−1^
*T* = 230 K
0.60 × 0.20 × 0.20 mm

#### Data collection

Bruker SMART 1K CCD diffractometer
Absorption correction: multi-scan (*SADABS*; Bruker, 2007 ▶) *T*_min_ = 0.832, *T*_max_ = 0.954
19429 measured reflections
3580 independent reflections
3283 reflections with *I* > 2σ(*I*)
*R*_int_ = 0.033

#### Refinement

*R*[*F* ^2^ > 2σ(*F* ^2^)] = 0.039
*wR*(*F* ^2^) = 0.084
*S* = 1.07
3580 reflections
257 parameters
1 restraint
H atoms treated by a mixture of independent and constrained refinement
Δρ_max_ = 0.28 e Å^−3^
Δρ_min_ = −0.38 e Å^−3^
Absolute structure: Flack (1983 ▶), 1681 Friedel pairs
Flack parameter: 0.02 (8)

### 

Data collection: *SMART* (Bruker, 2007 ▶); cell refinement: *SAINT* (Bruker, 2007 ▶); data reduction: *SAINT*; program(s) used to solve structure: *SHELXS97* (Sheldrick, 2008 ▶); program(s) used to refine structure: *SHELXL97* (Sheldrick, 2008 ▶); molecular graphics: *SHELXTL* (Sheldrick, 2008 ▶); software used to prepare material for publication: *SHELXTL*.

## Supplementary Material

Crystal structure: contains datablocks I, global. DOI: 10.1107/S1600536809023022/bi2377sup1.cif

Structure factors: contains datablocks I. DOI: 10.1107/S1600536809023022/bi2377Isup2.hkl

Additional supplementary materials:  crystallographic information; 3D view; checkCIF report

**Fig. 1.**
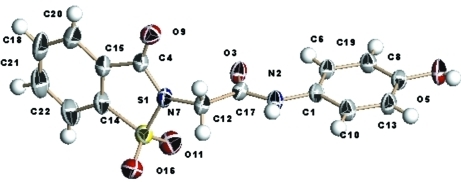
The asymmetric unit of the title compound. Displacement ellipsoids are drawn at the 50% probability level.

Enhanced figure: interactive version of Fig. 1

**Fig. 2.**
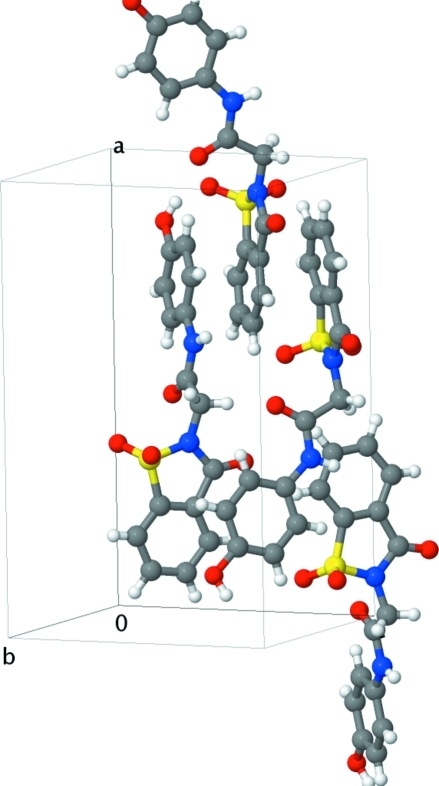
Perspective view of the contents of the unit cell showing the parallel stacking of the aromatic rings in layers perpendicular to the a axis.

Enhanced figure: interactive version of Fig. 2

## Figures and Tables

**Table 1:** Hydrogen-bond geometry (Å, °)

*D*—H⋯*A*	*D*—H	H⋯*A*	*D*⋯*A*	*D*—H⋯*A*
N10—H10⋯O14^i^	0.87 (3)	2.23 (3)	3.078 (3)	165 (3)
O14—H14⋯O9^ii^	0.82 (3)	1.91 (3)	2.725 (2)	173 (3)

Symmetry codes: (i) 
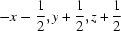
; (ii) 
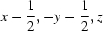
.

## supplementary crystallographic information

### Comment

Analgesics currently in use have high incidence of adverse reactions and can cause potentially lethal effects like hepatotoxicity and nephrotoxicity (Slattery *et al.*, 1996; McGoldrick & Bailie, 1997; Watkins *et al.*, 2006). A series of compounds bearing the acetaminophen (Tylenol) fragment linked to different lipophilic heterocyclic moieties were synthesized with a view to modulate its pharmacokinetic profile (Bazan & Alvarez-Builla, 1996*a*,*b*6; Vaccarino *et al.*, 2007). Of these new derivatives, the title compound (commonly called SCP-1) has a similar profile to that of acetaminophen but with shorter elimination half-life and clearance.

### Experimental

The title compound was synthesized following the procedure described by Vaccarino *et al.* (2007) and colourless needles suitable for X-ray analysis were obtained by recrystallization from an ethanol-water (8:1) mixture.

### Refinement

All H atoms were located in a difference density map and their positional parameters and *U*_iso_ included in the full-matrix least-squares refinement. Observed C—H bond lengths are in the range 0.91 (3)–0.97 (3) Å.

### Crystal data

**Table:**

C_15_H_12_N_2_O_5_S	*F*(000) = 688
*M**_r_* = 332.33	*D*_x_ = 1.405 Mg m^−^^3^
Orthorhombic, *P**n**a*2_1_	Mo *K*α radiation, λ = 0.71073 Å
Hall symbol: P 2c -2n	Cell parameters from 17706 reflections
*a* = 16.3588 (10) Å	θ = 2.5–30.5°
*b* = 9.6451 (6) Å	µ = 0.23 mm^−^^1^
*c* = 9.9603 (6) Å	*T* = 230 K
*V* = 1571.56 (17) Å^3^	Needle, colourless
*Z* = 4	0.60 × 0.20 × 0.20 mm

### Data collection

**Table:**

Bruker SMART 1K CCD diffractometer	3580 independent reflections
Radiation source: fine-focus sealed tube	3283 reflections with *I* > 2σ(*I*)
graphite	*R*_int_ = 0.033
φ and ω scans	θ_max_ = 27.5°, θ_min_ = 2.5°
Absorption correction: multi-scan (*SADABS*; Bruker, 2007)	*h* = −21→21
*T*_min_ = 0.832, *T*_max_ = 0.954	*k* = −12→12
19429 measured reflections	*l* = −12→12

### Refinement

**Table:**

Refinement on *F*^2^	Hydrogen site location: inferred from neighbouring sites
Least-squares matrix: full	H atoms treated by a mixture of independent and constrained refinement
*R*[*F*^2^ > 2σ(*F*^2^)] = 0.039	*w* = 1/[σ^2^(*F*_o_^2^) + (0.0183*P*)^2^ + 1.0724*P*] where *P* = (*F*_o_^2^ + 2*F*_c_^2^)/3
*wR*(*F*^2^) = 0.084	(Δ/σ)_max_ < 0.001
*S* = 1.07	Δρ_max_ = 0.28 e Å^−^^3^
3580 reflections	Δρ_min_ = −0.38 e Å^−^^3^
257 parameters	Extinction correction: *SHELXL97* (Sheldrick, 2008), Fc^*^=kFc[1+0.001xFc^2^λ^3^/sin(2θ)]^-1/4^
1 restraint	Extinction coefficient: 0.0063 (8)
Primary atom site location: structure-invariant direct methods	Absolute structure: Flack (1983), 1681 Friedel pairs
Secondary atom site location: difference Fourier map	Flack parameter: 0.02 (8)

### Special details

**Table:**

Geometry. All e.s.d.'s (except the e.s.d. in the dihedral angle between two l.s. planes) are estimated using the full covariance matrix. The cell e.s.d.'s are taken into account individually in the estimation of e.s.d.'s in distances, angles and torsion angles; correlations between e.s.d.'s in cell parameters are only used when they are defined by crystal symmetry. An approximate (isotropic) treatment of cell e.s.d.'s is used for estimating e.s.d.'s involving l.s. planes.
Refinement. Refinement of *F*^2^ against ALL reflections. The weighted *R*-factor *wR* and goodness of fit *S* are based on *F*^2^, conventional *R*-factors *R* are based on *F*, with *F* set to zero for negative *F*^2^. The threshold expression of *F*^2^ > σ(*F*^2^) is used only for calculating *R*-factors(gt) *etc*. and is not relevant to the choice of reflections for refinement. *R*-factors based on *F*^2^ are statistically about twice as large as those based on *F*, and *R*- factors based on ALL data will be even larger.

### Fractional atomic coordinates and isotropic or equivalent isotropic displacement parameters (Å^2^)

**Table:**

	*x*	*y*	*z*	*U*_iso_*/*U*_eq_	
S1	0.11997 (4)	0.20573 (6)	0.93772 (6)	0.03830 (15)	
O1	0.10058 (12)	0.3352 (2)	1.0007 (2)	0.0534 (5)	
N2	0.09100 (11)	0.0757 (2)	1.0375 (2)	0.0339 (4)	
O2	0.08966 (13)	0.1841 (2)	0.80485 (18)	0.0554 (5)	
C3	0.15368 (14)	0.0026 (2)	1.0986 (2)	0.0320 (5)	
O3	0.14167 (11)	−0.0882 (2)	1.17895 (19)	0.0451 (4)	
C4	0.31014 (15)	0.0065 (3)	1.0784 (3)	0.0467 (6)	
H4	0.3137 (16)	−0.067 (3)	1.135 (3)	0.034 (7)*	
C4A	0.23289 (14)	0.0552 (3)	1.0467 (2)	0.0353 (5)	
C5	0.37621 (17)	0.0677 (4)	1.0128 (4)	0.0608 (9)	
H5	0.430 (2)	0.035 (4)	1.032 (4)	0.081 (11)*	
C6	0.36573 (17)	0.1721 (4)	0.9215 (4)	0.0640 (9)	
H6	0.4123 (19)	0.209 (3)	0.873 (4)	0.065 (9)*	
C7A	0.22384 (13)	0.1609 (3)	0.9562 (3)	0.0398 (5)	
C7	0.2887 (2)	0.2225 (3)	0.8913 (3)	0.0561 (8)	
H7	0.283 (2)	0.295 (3)	0.830 (3)	0.058 (9)*	
C8	0.00639 (14)	0.0545 (3)	1.0733 (2)	0.0346 (5)	
H8B	−0.0203 (16)	0.141 (3)	1.072 (3)	0.038 (7)*	
H8A	0.0067 (16)	0.018 (3)	1.162 (3)	0.035 (7)*	
C9	−0.03588 (12)	−0.0469 (2)	0.9791 (2)	0.0298 (4)	
O9	0.00080 (9)	−0.11209 (18)	0.89225 (16)	0.0379 (4)	
N10	−0.11625 (11)	−0.0573 (2)	1.0033 (2)	0.0331 (4)	
H10	−0.1339 (17)	−0.008 (3)	1.070 (3)	0.045 (8)*	
C11	−0.17479 (12)	−0.1418 (2)	0.9368 (2)	0.0295 (4)	
C12	−0.25630 (14)	−0.1228 (3)	0.9725 (3)	0.0391 (6)	
H12	−0.2690 (17)	−0.049 (3)	1.033 (3)	0.047 (8)*	
C13	−0.31719 (13)	−0.2010 (3)	0.9129 (3)	0.0407 (6)	
H13	−0.3736 (19)	−0.187 (3)	0.933 (4)	0.066 (9)*	
C14	−0.29711 (13)	−0.2994 (2)	0.8175 (2)	0.0320 (5)	
O14	−0.35473 (10)	−0.3808 (2)	0.75619 (19)	0.0442 (5)	
H14	−0.397 (2)	−0.378 (3)	0.802 (4)	0.060 (10)*	
C15	−0.21627 (14)	−0.3179 (3)	0.7811 (2)	0.0341 (5)	
H15	−0.2007 (16)	−0.386 (3)	0.716 (3)	0.040 (7)*	
C16	−0.15511 (13)	−0.2396 (3)	0.8404 (2)	0.0320 (5)	
H16	−0.1000 (17)	−0.255 (3)	0.814 (3)	0.041 (7)*	

### Atomic displacement parameters (Å^2^)

**Table:**

	*U*^11^	*U*^22^	*U*^33^	*U*^12^	*U*^13^	*U*^23^
S1	0.0371 (3)	0.0407 (3)	0.0371 (3)	−0.0060 (2)	−0.0064 (3)	0.0063 (3)
O1	0.0547 (11)	0.0422 (10)	0.0633 (12)	0.0011 (9)	−0.0100 (10)	0.0011 (9)
N2	0.0253 (9)	0.0422 (11)	0.0343 (10)	−0.0052 (8)	−0.0035 (7)	0.0048 (9)
O2	0.0640 (12)	0.0651 (13)	0.0371 (10)	−0.0036 (11)	−0.0141 (9)	0.0105 (9)
C3	0.0288 (11)	0.0366 (12)	0.0306 (11)	−0.0023 (9)	−0.0040 (9)	−0.0026 (10)
O3	0.0430 (10)	0.0481 (11)	0.0443 (10)	0.0011 (8)	−0.0010 (8)	0.0133 (9)
C4	0.0312 (12)	0.0594 (17)	0.0496 (15)	0.0012 (12)	−0.0040 (11)	−0.0117 (14)
C4A	0.0278 (11)	0.0438 (13)	0.0343 (11)	−0.0037 (10)	−0.0027 (10)	−0.0065 (10)
C5	0.0260 (13)	0.081 (2)	0.075 (2)	−0.0045 (14)	−0.0007 (13)	−0.0239 (19)
C6	0.0398 (14)	0.081 (2)	0.072 (2)	−0.0244 (15)	0.0125 (16)	−0.009 (2)
C7A	0.0312 (11)	0.0480 (13)	0.0401 (14)	−0.0085 (10)	0.0011 (10)	−0.0019 (11)
C7	0.0491 (16)	0.065 (2)	0.0547 (18)	−0.0222 (15)	0.0078 (13)	0.0049 (15)
C8	0.0256 (10)	0.0460 (14)	0.0323 (12)	−0.0015 (10)	−0.0004 (9)	−0.0030 (11)
C9	0.0246 (9)	0.0372 (11)	0.0275 (10)	−0.0002 (9)	−0.0012 (8)	0.0020 (9)
O9	0.0238 (7)	0.0520 (10)	0.0379 (9)	−0.0013 (7)	0.0043 (6)	−0.0089 (8)
N10	0.0249 (9)	0.0425 (11)	0.0319 (10)	−0.0037 (8)	0.0045 (8)	−0.0087 (9)
C11	0.0240 (8)	0.0352 (10)	0.0293 (9)	−0.0040 (8)	0.0016 (9)	−0.0002 (10)
C12	0.0276 (10)	0.0465 (14)	0.0432 (14)	−0.0007 (10)	0.0078 (10)	−0.0145 (11)
C13	0.0219 (10)	0.0511 (13)	0.0492 (16)	−0.0017 (10)	0.0059 (10)	−0.0093 (12)
C14	0.0254 (10)	0.0402 (12)	0.0304 (11)	−0.0067 (9)	0.0012 (9)	0.0024 (9)
O14	0.0284 (9)	0.0613 (12)	0.0427 (10)	−0.0135 (9)	0.0038 (8)	−0.0150 (9)
C15	0.0296 (11)	0.0408 (13)	0.0319 (11)	−0.0023 (10)	0.0053 (9)	−0.0044 (10)
C16	0.0216 (10)	0.0401 (12)	0.0343 (12)	−0.0010 (9)	0.0055 (9)	−0.0010 (10)

### Geometric parameters (Å, °)

**Table:**

S1—O2	1.4286 (19)	C8—H8B	0.94 (3)
S1—O1	1.433 (2)	C8—H8A	0.95 (3)
S1—N2	1.668 (2)	C9—O9	1.226 (3)
S1—C7A	1.763 (2)	C9—N10	1.340 (3)
N2—C3	1.386 (3)	N10—C11	1.421 (3)
N2—C8	1.444 (3)	N10—H10	0.87 (3)
C3—O3	1.203 (3)	C11—C16	1.384 (3)
C3—C4A	1.484 (3)	C11—C12	1.392 (3)
C4—C4A	1.385 (3)	C12—C13	1.383 (3)
C4—C5	1.394 (4)	C12—H12	0.95 (3)
C4—H4	0.91 (3)	C13—C14	1.383 (3)
C4A—C7A	1.369 (4)	C13—H13	0.95 (3)
C5—C6	1.367 (5)	C14—O14	1.370 (3)
C5—H5	0.96 (4)	C14—C15	1.383 (3)
C6—C7	1.384 (4)	O14—H14	0.82 (3)
C6—H6	0.97 (3)	C15—C16	1.385 (3)
C7A—C7	1.377 (4)	C15—H15	0.96 (3)
C7—H7	0.93 (3)	C16—H16	0.95 (3)
C8—C9	1.522 (3)		
			
O2—S1—O1	117.12 (13)	N2—C8—H8B	108.3 (16)
O2—S1—N2	110.07 (11)	C9—C8—H8B	110.3 (16)
O1—S1—N2	109.35 (12)	N2—C8—H8A	106.1 (16)
O2—S1—C7A	113.30 (13)	C9—C8—H8A	109.9 (16)
O1—S1—C7A	112.41 (12)	H8B—C8—H8A	110 (2)
N2—S1—C7A	91.57 (11)	O9—C9—N10	124.6 (2)
C3—N2—C8	121.9 (2)	O9—C9—C8	122.84 (19)
C3—N2—S1	115.73 (16)	N10—C9—C8	112.52 (19)
C8—N2—S1	121.75 (17)	C9—N10—C11	128.3 (2)
O3—C3—N2	122.8 (2)	C9—N10—H10	115.0 (19)
O3—C3—C4A	128.6 (2)	C11—N10—H10	116.6 (19)
N2—C3—C4A	108.6 (2)	C16—C11—C12	119.3 (2)
C4A—C4—C5	117.2 (3)	C16—C11—N10	123.88 (19)
C4A—C4—H4	117.7 (17)	C12—C11—N10	116.8 (2)
C5—C4—H4	125.0 (17)	C13—C12—C11	120.6 (2)
C7A—C4A—C4	120.1 (2)	C13—C12—H12	121.3 (17)
C7A—C4A—C3	112.9 (2)	C11—C12—H12	117.9 (17)
C4—C4A—C3	127.0 (2)	C12—C13—C14	119.9 (2)
C6—C5—C4	121.8 (3)	C12—C13—H13	122 (2)
C6—C5—H5	120 (2)	C14—C13—H13	118 (2)
C4—C5—H5	119 (2)	O14—C14—C15	117.9 (2)
C5—C6—C7	121.2 (3)	O14—C14—C13	122.4 (2)
C5—C6—H6	120.3 (19)	C15—C14—C13	119.7 (2)
C7—C6—H6	118 (2)	C14—O14—H14	108 (2)
C4A—C7A—C7	123.2 (2)	C14—C15—C16	120.6 (2)
C4A—C7A—S1	110.83 (17)	C14—C15—H15	121.2 (16)
C7—C7A—S1	126.0 (2)	C16—C15—H15	118.1 (16)
C7A—C7—C6	116.6 (3)	C11—C16—C15	119.9 (2)
C7A—C7—H7	123 (2)	C11—C16—H16	121.3 (16)
C6—C7—H7	120 (2)	C15—C16—H16	118.8 (16)
N2—C8—C9	111.95 (19)		
			
O2—S1—N2—C3	121.36 (18)	O2—S1—C7A—C7	63.0 (3)
O1—S1—N2—C3	−108.64 (18)	O1—S1—C7A—C7	−72.6 (3)
C7A—S1—N2—C3	5.82 (19)	N2—S1—C7A—C7	175.7 (3)
O2—S1—N2—C8	−67.6 (2)	C4A—C7A—C7—C6	0.1 (5)
O1—S1—N2—C8	62.4 (2)	S1—C7A—C7—C6	179.4 (2)
C7A—S1—N2—C8	176.87 (19)	C5—C6—C7—C7A	−0.9 (5)
C8—N2—C3—O3	4.5 (4)	C3—N2—C8—C9	−96.0 (3)
S1—N2—C3—O3	175.5 (2)	S1—N2—C8—C9	93.5 (2)
C8—N2—C3—C4A	−176.0 (2)	N2—C8—C9—O9	6.7 (3)
S1—N2—C3—C4A	−5.0 (2)	N2—C8—C9—N10	−174.0 (2)
C5—C4—C4A—C7A	−1.1 (4)	O9—C9—N10—C11	−0.2 (4)
C5—C4—C4A—C3	177.2 (3)	C8—C9—N10—C11	−179.5 (2)
O3—C3—C4A—C7A	−179.5 (3)	C9—N10—C11—C16	6.3 (4)
N2—C3—C4A—C7A	1.0 (3)	C9—N10—C11—C12	−173.7 (2)
O3—C3—C4A—C4	2.1 (4)	C16—C11—C12—C13	0.3 (4)
N2—C3—C4A—C4	−177.4 (2)	N10—C11—C12—C13	−179.8 (2)
C4A—C4—C5—C6	0.3 (4)	C11—C12—C13—C14	0.3 (4)
C4—C5—C6—C7	0.7 (5)	C12—C13—C14—O14	179.3 (2)
C4—C4A—C7A—C7	0.9 (4)	C12—C13—C14—C15	−0.7 (4)
C3—C4A—C7A—C7	−177.6 (3)	O14—C14—C15—C16	−179.4 (2)
C4—C4A—C7A—S1	−178.4 (2)	C13—C14—C15—C16	0.6 (4)
C3—C4A—C7A—S1	3.1 (3)	C12—C11—C16—C15	−0.4 (3)
O2—S1—C7A—C4A	−117.6 (2)	N10—C11—C16—C15	179.7 (2)
O1—S1—C7A—C4A	106.8 (2)	C14—C15—C16—C11	−0.1 (4)
N2—S1—C7A—C4A	−5.0 (2)		

### Hydrogen-bond geometry (Å, °)

**Table:**

*D*—H···*A*	*D*—H	H···*A*	*D*···*A*	*D*—H···*A*
N10—H10···O14^i^	0.87 (3)	2.23 (3)	3.078 (3)	165 (3)
O14—H14···O9^ii^	0.82 (3)	1.91 (3)	2.725 (2)	173 (3)

Symmetry codes: (i) −*x*−1/2, *y*+1/2, *z*+1/2; (ii) *x*−1/2, −*y*−1/2, *z*.
